# Effect of age on stress induced changes in aortic distensibility

**DOI:** 10.1186/1532-429X-11-S1-O60

**Published:** 2009-01-28

**Authors:** Haroon L Chughtai, William G Hundley, Tim Morgan, Craig Hamilton

**Affiliations:** Wake Forest University Health Sciences, Winston Salem, NC USA

**Keywords:** Cardiovascular Magnetic Resonance, Cardiac Magnetic Resonance, Dobutamine, Heart Rate Response, Aortic Stiffness

## Introduction

Aging increases aortic stiffness. We describe a novel method for assessing aortic distensibility throughout stress with dobutamine.

## Purpose

Previous studies have shown aortic stiffness to be a predictor of adverse cardiovascular outcomes and mortality. Aging is one of the most significant factors associated with aortic stiffness. Aortic stiffness can be assessed by measuring aortic distensibility non-invasively with cardiac magnetic resonance. We undertook this study to assess and understand the changes in aortic distensibility with stress (induced by dobutamine) and find an association of these changes with advancing age.

## Methods

We studied 128 patients (48% women) aged 55–85 years in which aortic distensibility was determined during intravenous dobutamine infused to achieve 85% of predicted heart rate response for age. Image acquisition was accomplished with phase-contrast gradient echo cardiovascular magnetic resonance. Images were acquired perpendicular to the course of ascending aorta at the middle level of the left atrium. Aortic distensibility was determined by measuring cardiac cycle dependent changes in aortic area/{brachial pulse pressure x the end-diastolic area of the aorta}. Changes between different stages of stress were calculated and correlated with age using linear regression.

## Results

**Figure 1:**
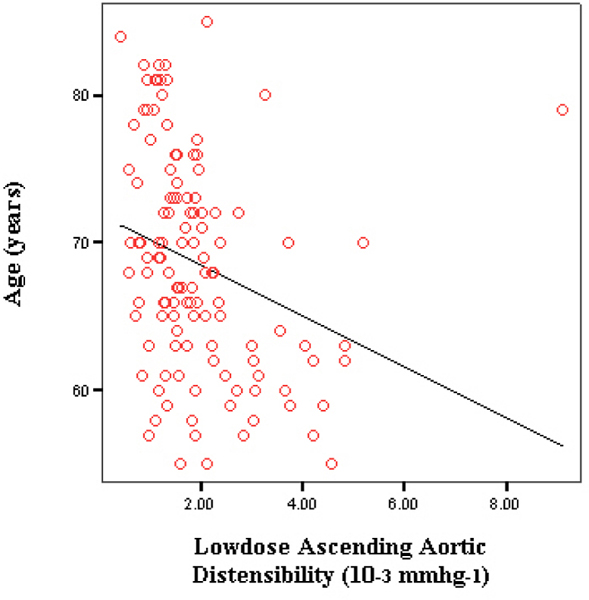
Figure 1

**Figure 2:**
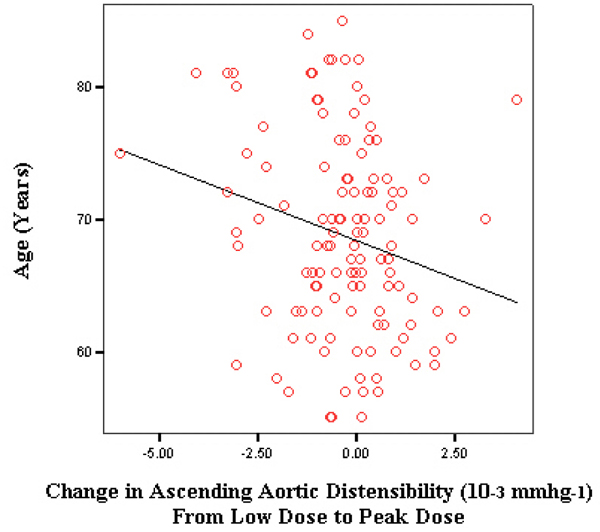
Figure 2

Resting baseline ascending aortic distentesibility was 2.0 × 10^-3^ mmhg^-1^. After infusion of low dose dobutamine (7.5 μg/kg/min), mean ascending aortic distensibility was 1.9 × 10^-3^ mmhg^-1^. At peak stress, mean ascending aortic distensibility was 1.8 × 10^-3^ mmhg^-1^. On Pearson correlation, all the individual distensibilities (baseline, low dose and peak dose) correlated negatively with age (baseline and low dose significant at p of .00). In the second step, differences between each of baseline to low dose and low dose to high dose distensibilities were calculated. From baseline to low dose, mean difference was 0.178 × 10^-3^ mmhg^-1^ which correlated with age at an r of -0.24 (significant at a p value of .00). The mean difference between low dose to peak dose was -0.308 × 10^-3^ mmhg^-1^ which correlated with age at an r of -0.22 (significant at a p value of .01). A partial correlation was also done after adjustment for gender, hypertension and diabetes, which showed persistence of negative correlation of baseline, low dose and peak dose ascending aortic distensibilities with age (baseline and low dose were statistically significant). The adjusted analysis also showed the independent negative correlation of age with change in ascending aortic distensibility from low dose to peak dose (r of -0.25 at a p value of .01). Figures 1 and 2.

## Conclusion

Our study indicates that advanced age is an independent predictor of impaired ascending aortic distensibility and inability to adequately change distensibility during stress. These data imply that advanced aging may alter the relationship between aortic stiffness and left ventricular emptying during pharmacologic stress.

